# The Effect of Silymarin on the Prevention of Atrial Fibrillation After Coronary Artery Bypass Grafting

**DOI:** 10.21470/1678-9741-2023-0422

**Published:** 2024-11-18

**Authors:** Bogdan Okiljevic, Ranko Zdravkovic, Andrej Preveden, Mihaela Preveden, Nikola Mladenovic, Stamenko Susak

**Affiliations:** 1 Cardiac Surgery Department, Institute for Cardiovascular Diseases Dedinje, Belgrade, Serbia; 2 Cardiac Surgery Department, Institute for Cardiovascular Diseases of Vojvodina, Sremska Kamenica, Serbia; 3 Faculty of Medicine, University of Novi Sad, Novi Sad, Serbia

**Keywords:** Atrial Fibrilation, C-Reactive Protein, Aspartate Aminotransferases, Erythrocyte Transfusion, Coronary Artery Bypass, Control Groups

## Abstract

**Introduction:**

Postoperative atrial fibrillation is a frequent complication after coronary artery bypass grafting and is associated with increased mortality. The effects of various drugs on the occurrence of postoperative atrial fibrillation have been studied, but no one has looked into the effect of silymarin on the occurrence of postoperative atrial fibrillation.

**Methods:**

This prospective experimental study included 160 patients undergoing coronary artery bypass grafting. The experimental group received 400 mg of silymarin orally three days before the surgery, while the control group did not. The occurrence of postoperative atrial fibrillation was monitored. Patients’ clinical data and postoperative characteristics were investigated to elucidate their impact on postoperative atrial fibrillation.

**Results:**

Postoperative atrial fibrillation occurred in significantly fewer patients in the experimental group (14 vs. 30, P=0.008). There were also lower mean values of postoperatively measured C-reactive protein (P<0.0005) and aspartate aminotransferase (P=0.001) in the experimental group. Within the multivariate regression model, a non-silymarin group (odds ratio 0.296 [0.109-0.807], P=0.005), postoperative red blood cell transfusion (odds ratio 5.218 [1.930-14.107], P=0.001), left atrial diameter (odds ratio 7.800 [2.122-28.672], P=0.002), postoperative C-reactive protein (odds ratio 1.020 [1.008-1.032], P=0.001), and CHA_2_DS_2_-VASc score (standing for congestive heart failure, hypertension, age ≥ 75 years [doubled], diabetes, stroke [doubled], vascular disease, age 65 to 74 years, and sex category [female]) (odds ratio 1.873 [1.279-2.743], P=0.001) proved to be independently associated with the development of postoperative atrial fibrillation.

**Conclusion:**

This study showed that preoperative administration of silymarin significantly reduces the development of atrial fibrillation after coronary artery bypass grafting.

## INTRODUCTION

**Table:** 

Abbreviations, Acronyms & Symbols				
ALT	= Alanine aminotransferase		EuroSCORE	= European System for Cardiac Operative Risk Evaluation
AST	= Aspartate aminotransferase		ICU	= Intensive care unit
BMI	= Body mass index		IL	= Interleukin
CABG	= Coronary artery bypass grafting		IQR	= Interquartile range
CHA_2_DS_2_-VASc	= Congestive heart failure, hypertension, age ≥ 75 years (doubled), diabetes, stroke (doubled), vascular disease, age 65 to 74 years, and sex category (female)		LA LVEF NYHA OR	= Left atrial = Left ventricular ejection fraction = New York Heart Association = Odds ratio
CI	= Confidence interval		POAF	= Postoperative atrial fibrillation
CK	= Creatin kinase		RBC	= Red blood cell
COPD	= Chronic obstructive pulmonary disease		SD	= Standard deviation
CPB	= Cardiopulmonary bypass		VKA	= Vitamin K antagonists
CRP	= C-reactive protein		WBC	= White blood cells
ECG	= Electrocardiography			

Atrial fibrillation after cardiac surgery occurs in 30-60% of patients, mainly in the first two to four days after surgery^[1-6]^. Although postoperative atrial fibrillation (POAF) is mostly self-limiting and most of it is converted to sinus rhythm during hospitalization, it has been shown to be associated with increased mortality as well as numerous complications, including multi-organ failure, stroke, hemodynamic instability, and thromboembolism^[2,7,8]^. All these lead to prolonged hospitalization and increased hospital costs^[9]^. Although there have been various strategies to reduce the incidence, POAF is still a very common complication in cardiac surgery.

The exact mechanism of occurrence is insufficiently known, but it is considered that the main etiological factors are inflammation, oxidative stress caused by ischemia/reperfusion injury, autonomic nervous system dysfunction, and structural changes in the heart^[2,3,10]^. A large meta-analysis showed a correlation between the values of C-reactive protein (CRP) and interleukins (IL) 6, 8, and 10 and the occurrence of POAF^[2]^. The CRP/albumin ratio, a novel parameter of inflammation, has been shown to be superior to CRP or albumin levels alone in determining inflammatory status in several cardiovascular diseases, and a recent study showed a correlation between this ratio and the occurrence of POAF^[11]^. This leads to the conclusion that anti-inflammatory and antioxidant therapy could play a role in the prevention of POAF.

Silymarin is extracted from the seeds of *Silybum marianum* (L.) Gaertn, also known as Mary thistle and milk thistle^[12]^. It is registered as a traditional herbal medicine and as a hepatoprotective agent. It potentiates the effects of the physiological antioxidants (glutathione, superoxide dismutase) and prevents the reduction of their concentrations, as well as its structural and functional consequences^[13]^. The aim of this study was to examine the effect of preoperative silymarin administration on the occurrence of atrial fibrillation after coronary artery bypass grafting (CABG).

## METHODS

### Study Population

The study protocol complied with the Declaration of Helsinki and was approved by the Ethics Committee of the Institute for Cardiovascular Diseases of Vojvodina, Serbia (approval number 313-1/12). It is registered with ClinicalTrials.gov NCT06114719. The procedures were performed after obtaining the informed written consent from the patients. *A priori*, the study power analysis, based on the literature data^[14]^, determined a minimum of 144 patients (1:1 case/control ratio), to be able to reject the null hypothesis with probability 0.95 and the type I error probability 0.05. A total of 160 patients of both sexes (ages 46-84 years) scheduled for elective CABG using cardiopulmonary bypass (CPB) were included in this prospective, experimental study. The study was conducted at the Clinic for Cardiovascular Surgery of the Institute for Cardiovascular Diseases of Vojvodina between February 2021 and March 2022. The study included patients who underwent their first cardiac surgery, with a left ventricular ejection fraction (LVEF) > 35% and less than moderate mitral regurgitation. Patients with preoperative atrial fibrillation, previous history of interventionally treated arrhythmias, end-stage renal disease requiring hemodialysis, and chronic inflammatory and neoplastic diseases were excluded from the study. The fulfillment of the criteria for inclusion in the study was done on the basis of insight into the electronic card of the patient.

### Intervention

Patients were divided into two groups (experimental and control) with 80 patients each. Patients who were hospitalized at least three days before the scheduled operation were classified in the experimental group, while the rest were classified in the control group. Subjects of the experimental group received silymarin (capsules containing 100 mg silymarin, expressed as silibinin) three days preoperatively. The dose they received was the recommended dose for the registered indications (400 mg daily, orally, divided into two doses).

### Anesthesia and Surgical Technique

Anesthesia was induced with combinations of sufentanil, midazolam, propofol, and rocuronium bromide. After intubation, the lungs were mechanically ventilated with an oxygen/air mixture of 50:50. Anesthesia was maintained with sevoflurane, analgesia with a continuous infusion of sufentanil, and muscle relaxation with intermittent administration of rocuronium bromide. Perioperative and postoperative monitoring included continuous arterial and central venous pressure measurement, electrocardiography (ECG), oxygen saturation (pulse oximetry), body temperature measured in the nasopharynx, and diuresis. Arterial blood gas analyses were performed intermittently.

In our center, all CABG operations are performed through a full midline sternotomy. After sternotomy, the left internal mammary artery was harvested extrapleuraly in a skeletonized fashion (there was no persistent communication between the pericardial and pleural spaces). The great saphenous vein was simultaneously (in skeletonized fashion) harvested through continuous incision. Heparin was administered to keep the activated clotting time > 480 s. Aortic and double-stage venous cannulation were performed. The CPB circuit was standard, with mild hypothermia (32-34°C) and antegrade intermittent cold (extracellular crystalloid or blood) cardioplegia. All distal and proximal anastomoses were constructed during a single cross-clamping period. After the conclusion of CPB, two drains (retrocardial and retrosternal) were used with negative pressure suction. Sternum and wounds were closed in a standard manner, without pericardial closure.

### Demographic, Clinical, and Laboratory Measures

The following data were registered: age, sex, body mass index, comorbidities (hypertension, smoking, chronic obstructive pulmonary disease, diabetes mellitus, peripheral arterial occlusive disease, history of myocardial infarction, history of cerebrovascular stroke), hemoglobin, white blood cells (WBC), CRP, creatinine clearance (Cockroft-Gault calculator), left atrial (LA) size (measured by transthoracic echocardiography as latero-lateral diameter in the long parasternal axis), LVEF, CHA_2_DS_2_-VASc (standing for congestive heart failure, hypertension, age ≥ 75 years [doubled], diabetes, stroke [doubled], vascular disease, age 65 to 74 years, and sex category [female]) score, New York Heart Association (NYHA) classification, and intraoperative parameters (aortic cross-clamping time, CPB time, type of cardioplegic solution, CABG number). Among the postoperative parameters, the following were registered: duration of mechanical ventilation (in hours), red blood cell (RBC) transfusion, use of inotropes, WBC, CRP, creatine kinase (CK), CK-MB, aspartate aminotransferase (AST), alanine aminotransferase (ALT), use of vitamin K antagonists (VKA), the intensive care unit (ICU) stay, presence of pericardial effusion (over 0.5 cm separation of pericardial sheets), readmission to the ICU, stroke, or in-hospital deaths.

### Outcomes

The occurrence of POAF after CABG was the primary outcome. In this study, POAF was defined as any dysrhythmia that represents the ECG characteristics of atrial fibrillation lasting at least 30 s on a rhythm strip or 12-lead ECG^[15]^. Patients from both groups were continuously monitored postoperatively in the ICU, as well as later in the semi-ICU for the first four postoperative days. A 12-channel ECG was performed daily in the morning, as well as after each episode of dyspnea, chest pain, and palpitations.

Secondary outcomes included postoperative values of WBC and CRP, length of stay in the ICU, length of hospitalization, and occurrence of postoperative complications (pericardial effusion, stroke, in-hospital death).

### Statistical Analyses

Descriptive statistics measures were used: arithmetic mean, standard deviation, median, quartiles, frequencies, and percentages. For numeric variables, the *t*-test for independent samples and the Mann-Whitney test were used to detect differences between the experimental and control groups. The correlation of categorical variables was examined using the Chi-squared test for contingency tables or using the Fisher’s exact test. A two-way analysis of variance was performed to determine the significance of interaction between the independent variables on the dependent variable. The influence of variables on the treatment outcome was determined using univariate and multivariate binary logistic analysis. A *P*<0.05 value was taken for the statistical significance of the test. Statistical analysis was performed using IBM Corp. Released 2010, IBM SPSS Statistics for Windows, Version 19.0, Armonk, NY: IBM Corp.

## RESULTS

### Baseline Characteristics of the Population

As shown in Table 1, there were no statistically significant differences in patient baseline characteristics (age, sex, comorbidities, NYHA class), scores (European System for Cardiac Operative Risk Evaluation II and CHA_2_DS_2_-VASc), echocardiography findings (LVEF, diastolic dysfunction, LA diameter), and preoperative laboratory findings (hemoglobin, WBC, CRP) between the control and experimental group.

**Table 1:** Baseline characteristics, echocardiography findings, and laboratory analysis.

Characteristics	Control group (n = 80)	Experimental group (n = 80)	*P*-value
Male, n (%)	68 (85.0)	63 (78.8)	0.41
Age (years), mean ± SD	66.2 ± 8.2	64.1 ± 8.7	0.12
BMI (kg/m^2^), mean ± SD	29.1 ± 3.9	28.5 ± 3.9	0.30
Smoking history, n (%)	48 (60.0)	42 (52.5)	0.43
Hypertension, n (%)	80 (100)	80 (100)	
COPD, n (%)	20 (25.0)	25 (31.3)	0.48
Diabetes mellitus, n (%)	17 (21.3)	27 (33.8)	0.11
Peripheral artery disease, n (%)	15 (18.8)	20 (25.0)	0.44
Previous myocardial infarction, n (%)	42 (52.5)	48 (60.0)	0.43
Stroke history (%)	7 (8.8)	5 (6.3)	0.765
EuroSCORE II	0.9 (0.7-1.8)	1.1 (0.8-1.7)	0.49
NYHA class, n (%)			
I-II	75 (93.7)	73 (91.2)	0.38
III-IV	5 (6.3)	7 (8.8)	
Preoperative creatinine clearance, n (%)			
Normal (> 85 ml/min)	68 (85.0)	64 (80.0)	0.438
Moderately impaired (50-85 ml/min)	10 (12.5)	15 (18.8)	
Severely impaired (< 50 ml/min)	2 (2.5)	1 (1.3)	
CHA_2_DS_2_-VASc score, n (%)			
0-2	26 (32.5)	24 (30.0)	0.417
3-4	44 (55.0)	44 (55.0)	
> 4	10 (12.5)	12 (15.0)	
LVEF (%)	57 (50.0-60.0)	55 (47.5-60)	0.106
Left atrial diameter (cm), mean ± SD	3.8 ± 0.4	3.8 ± 0.4	0.53
Diastolic dysfunction (grade), n (%)			
0	17 (21.3)	15 (18.8)	0.77
1	59 (73.8)	59 (73.8)	
2	4 (5.0)	6 (7.5)	
3-4	0 (0)	0 (0)	
Preoperative hemoglobin (gr/L)	141.0 (130.0-150.5)	141 (132.5-146.0)	0.93
Preoperative WBC (× 10^9^/L)	6.8 (5.6-8.1)	7.3 (6.4-8.8)	0.12
Preoperative CRP value (mg/L)	3.3 (2.4-4.8)	3.3 (2.1-5.8)	0.56

Categorical data are presented as counts and percentages, continuous data as medians and interquartile ranges

BMI=body mass index; CHA_2_DS_2_-VASc=congestive heart failure, hypertension, age ≥ 75 years (doubled), diabetes, stroke (doubled), vascular disease, age 65 to 74 years, and sex category (female); COPD=chronic obstructive pulmonary disease; CRP=C-reactive protein; EuroSCORE=European System for Cardiac Operative Risk Evaluation; LVEF=left ventricular ejection fraction; NYHA=New York Heart Association; SD=standard deviation; WBC=white blood cells

### Perioperative and Postoperative Characteristics

Table 2 shows surgery-related factors and ICU management data. There were no statistically significant differences in the number of grafted coronary arteries, type of cardioplegic solution used, aortic cross-clamping time, or CPB time between the groups. The duration of mechanical ventilation and the number of patients that needed inotropes postoperatively were similar in both groups. Two patients were readmitted to the ICU, both from the control group (no statistically significant differences between the groups in the rates of ICU readmission were observed). There was statistically significant (*P*<0.0005) difference in the mean ICU stay between the groups. The mean ICU stay was shorter in the experimental group, with a median of one day (interquartile range [IQR] 1-2) than in the control group (two days, IQR 2-2).

**Table 2:** Intraoperative data and ICU management.

Characteristics	Control group (n = 80)	Experimental group (n = 80)	*P*-value
Number of CABG, n (%)			
1	3 (3.8)	1 (1.2)	0.65
2	25 (31.2)	28 (35.0)	
3	35 (43.8)	41 (51.3)	
4	10 (12.5)	9 (11.3)	
5	7 (8.7)	1 (1.2)	
CPB time (min)	66 (55-82)	62 (53-76)	0.18
Aortic cross-clamping time (min)	60.0 (49.0-75.0)	56.5 (47.5-67.0)	0.25
Type of cardioplegic solution, n (%)			
Cold crystalloid	63 (78.8)	67 (83.8)	0.54
Cold blood	17 (21.3)	13 (16.3)	
Mechanical ventilation duration (hours)	9.5 (8.0-15.0)	9.0 (8.0-13.0)	0.22
Need for inotropes, n (%)	32 (40.0)	33 (41.3)	1
Readmission to the ICU, n (%)	2 (2.5)	0 (0)	0.50
ICU stay (days)	2.0 (2.0-2.0)	1.0 (1.0-2.0)	< 0.0005

Categorical data are presented as counts and percentages, continuous data as medians and interquartile ranges

CABG=coronary artery bypass grafting; CPB=cardiopulmonary bypass; ICU=intensive care unit

Table 3 summarizes postoperative findings and the incidence of complications between the groups. The rate of new-onset atrial fibrillation was statistically significantly lower in the experimental (17.5%) than in the control group (37.5%) (*P*=0.008). There were also lower mean values of postoperatively measured CRP (*P*<0.0005) and AST (*P*=0.001) in the experimental group. The incidence of pericardial effusion, RBC transfusion rates, CK, CK-MB, and ALT were similar in both groups. Twenty patients (12.5%) were discharged on VKA, eleven from the control and nine from the experimental group. In our study, stroke was an infrequent complication, with incidence of 1.25% (2/160). One patient developed a stroke intraoperatively, but the second developed a stroke on the fourth postoperative day due to an episode of atrial fibrillation. There were two (1.25%) in-hospital deaths, one due to a massive intraoperative stroke and the second one due to acute necrotizing pancreatitis. The mean hospital stay was shorter in the experimental group (7 *vs.* 8 days; *P*<0.0005).

**Table 3:** Postoperative data and complications.

Characteristics	Control group (n = 80)	Experimental group (n = 80)	*P*-value
New-onset POAF, n (%)	30 (37.5)	14 (17.5)	0.008
Postoperative pericardial effusion, n (%)	6 (7.5)	13 (16.3)	0.14
Postoperative RBC transfusion, n (%)	43 (53.8)	42 (52.5)	1.0
Postoperative CK (IU/L)	408.5 (283.5-681.5)	388.5 (295.0-517.5)	0.37
Postoperative, CK-MB (IU/L)	24.0 (20.0-33.5)	24.0 (17.0-30.0)	0.14
Postoperative AST (U/L)	35.5 (29.0-64.5)	31.0 (26.0-31.0)	0.001
Postoperative ALT (U/L)	28.0 (20.0-41.0)	28.0 (22.0-33.0)	0.64
Postoperative WBC (× 10^9^/L)	13.0 ± 3.1	12.7 ± 3.3	0.52
Postoperative CRP (mg/L)	164.2 (128.0-188.8)	124.7 (100.4-144.6)	< 0.0005
Discharge with VKA, n (%)	11 (13.8)	9 (11.3)	0.81
Postoperative stroke, n (%)	2 (2.5)	0 (0)	0.497
In-hospital death, n (%)	2 (2.5)	0 (0)	0.497
Hospital stay (days)	8.0 (7.0-8.0)	7.0 (6.0-8.0)	< 0.0005

ALT=alanine aminotransferase; AST=aspartate aminotransferase; CK=creatine kinase; CRP=C-reactive protein; POAF=postoperative atrial fibrillation; RBC=red blood cell; VKA=vitamin K antagonist; WBC=white blood cells

Patients who developed POAF after CABG were older than patients without POAF (*P*=0.004), with a higher CHA_2_DS_2_-VASc mean score (*P*<0.0005) and LA diameter mean value (*P*=0.009) (Table 4). They had longer mean mechanical ventilation time and postoperative CRP levels (*P*<0.0005). POAF is associated with longer ICU and hospital stays (*P*<0.0005). Neither postoperative CRP level (F=2.041, *P*=0.155), nor LA diameter (F=1.258, *P*=0.235), nor their combination (F=0.106, *P*=0.745) had significant interaction with the effect of silymarin use on the occurrence of POAF.

**Table 4:** Patient characteristics, findings, and complication according to occurrence of POAF.

Characteristics	Non-POAF	POAF	*P*-value
n (%)	116 (72.5)	44 (27.5)	
Age (years), mean ± SD	64.0 ± 8.3	68.3 ± 8.2	0.004
Left atrial diameter (cm), mean ± SD	3.7 ± 0.4	3.9 ± 0.4	0.009
Preoperative creatinine clearance, n (%)			
Normal (> 85 ml/min)	98 (74.2)	34 (25.8)	0.017
Moderately impaired (50-85 ml/min)	18 (72)	7 (28)	
Severely impaired, (< 50 ml/min)	0 (0)	3 (100)	
CHA_2_DS_2_-VASc score	3.0 (2.0-4.0)	4.0 (3.0-5.0)	< 0.0005
Mechanical ventilation duration (hours)	9.0 (8.0-13.0)	11.5 (9.0-16.0)	< 0.0005
Postoperative RBC transfusion, n (%)			
Yes	53 (62.4)	12 (16)	0.004
No	63 (84)	32 (37.6)	
Postoperative CRP (mg/L)	129.8 (108.8-161.0)	172.78 (129.0-192.1)	< 0.0005
ICU stay (days)	1.0 (1.0-2.0)	2.0 (2.0-2.0)	< 0.0005
Hospital stay (days)	7.0 (7.0-8.0)	8.0 (7.0-9.0)	< 0.0005

CHA_2_DS_2_-VASc=congestive heart failure, hypertension, age ≥ 75 years (doubled), diabetes, stroke (doubled), vascular disease, age 65 to 74 years, and sex category (female); CRP=C-reactive protein; ICU=intensive care unit; POAF=postoperative atrial fibrillation; RBC=red blood cell; SD=standard deviation

### Regression Analyses

Univariate predictors of POAF were group (experimental group had 65% lower odds), postoperative RBC transfusion, age, LA dimension, postoperative CRP, and CHA_2_DS_2_-VASc score (Table 5). Multivariate logistic regression analysis showed that independent predictors of POAF were group (the experimental group had 70% lower odds), postoperative RBC transfusion, LA dimension, postoperative CRP, and CHA_2_DS_2_-VASc score.

**Table 5:** Results of univariate and multivariate analysis of predictors of POAF.

Variable	Univariate regression analysis		Multivariate regression analysis	
	OR (95% CI)	*P*-value	OR (95% CI)	*P*-value
Group (experimental *vs.* control)	0.354 (0.170-0.736)	0.005	0.296 (0.109-0.807)	0.017
Postoperative RBC transfusion	3.170 (1.486-6.760)	0.003	5.218 (1.930-14.107)	0.001
Age	1.067 (1.020-1.116)	0.005	/	Non-significant
Left atrial diameter	3.337 (1.327-8.390)	0.01	7.800 (2.122-28.672)	0.002
Postoperative CRP	1.020 (1.010-1.029)	< 0.0005	1.020 (1.008-1.032)	0.001
CHA_2_DS_2_-VASc score	1.851 (1.368-2.505)	< 0.0005	1.873 (1.279-2.743)	0.001

CHA_2_DS_2_-VASc=congestive heart failure, hypertension, age ≥ 75 years (doubled), diabetes, stroke (doubled), vascular disease, age 65 to 74 years, and sex category (female); CI=confidence interval; CRP=C-reactive protein; OR=odds ratio; POAF=postoperative atrial fibrillation; RBC=red blood cell

## DISCUSSION

CABG is the standard of care for the treatment of advanced coronary artery disease. Despite its importance, this operation is associated with a risk of postoperative cardiac as well as non-cardiac complications. POAF is the most common cardiac complication after CABG. It is not only associated with increased postoperative mortality but also with a significant decrease in long-term survival rate. In the study by El-Chami et al., 169 consecutive patients underwent isolated CABG at a single center, survival rates at 10 years after surgery were 55% in those with POAF requiring treatment, compared with 70% without POAF^[16]^. Despite a good response to therapy and a variety of treatment options for this common arrhythmia, pre-diagnosis of POAF as well as prophylactic therapy could prevent potential complications and morbidities, lower health-care costs, mortality rates, and length of stay in ICUs and hospitals^[2]^. So far, meta-analyses of randomized studies of the use of amiodarone, statins, ꞵ-blockers, calcium blockers, and angiotensin-converting enzyme inhibitors have been published^[17]^. Also interesting are randomized studies by Ozaydin^[18]^ and Soleimani^[14]^ that showed that treatment with N-acetylcysteine preoperatively and immediately postoperatively contributes to a lower incidence of POAF, as well as a meta-analysis by Polymeropoulos^[19]^ on the effectiveness of vitamin C.

Although the pathogenesis of POAF remains uncertain, accumulating evidence suggests an important role of inflammatory mechanisms and mediators^[2,3,10]^. Such inflammation affects atrial conduction during atrial fibrillation by changing sodium channel function through the reduction of sodium currents and consequent upstroke velocity^[20]^. Evidence suggests that CPB-induced inflammation is interconnected with atrial remodeling, which is associated with the development of atrial fibrillation^[14]^. Oxidative stress is caused by an increase in reactive oxygen species and is associated with a more oxidized cellular redox state, as measured by the loss of glutathione^[21]^. High amounts of reactive oxygen species can cause deoxyribonucleic acid damage, apoptosis, and myocyte dysfunction^[22,23]^.

We evaluated the use of silymarin on the occurrence of atrial fibrillation after CABG. Silymarin, in addition to its well-known hepatoprotective effect, also has anti-inflammatory and antioxidant effects^[24,25]^. Silymarin contributes to antioxidant defenses in various ways. First, by direct radical scavenging. Second, by preventing the free formation of radical materials by inhibiting specific enzymes, producing free radical materials, or maintaining integrity in stress conditions in the mitochondrial electron transportation chain. Third, it contributes mainly to the preservation of the cell's optimal redox state by triggering several antioxidant enzymes and non-enzymatic antioxidants^[26]^. On the other hand, silymarin has a good safety profile^[27]^. Numerous animal studies have shown the cardioprotective effect of silymarin based on various mechanisms^[12]^. A study that looked at the effect of preoperative silymarin administration at a dose of 140 mg three times daily three days before elective CABG found that it reduced CRP, IL-1, IL-6, and tumor necrosis factor significantly^[23]^. Based on that, we came up with the hypothesis that silymarin treatment before surgery, through anti-inflammatory and antioxidant effects, would reduce the incidence of POAF. To our knowledge, this is the first study to examine the association between silymarin treatment and the occurrence of POAF after cardiac surgery. The most significant result of this study was that patients who received silymarin three days before surgery at a dose of 400 mg daily had a significantly lower incidence of POAF.

### Limitations

This study has some limitations. First of all, we used a therapeutic dose of silymarin for a short period of time. Some future studies could examine the efficacy and safety of higher doses of silymarin as well as a longer period of use before surgery. The study included a relatively small number of patients. For more significant conclusions, a multicenter study should be conducted, which would include a large number of patients. Finally, the results of this study could be influenced by some unknown factors that are not included in the study.

## CONCLUSION

This is the first study to examine the association between silymarin treatment and the occurrence of POAF after CABG. We could say that silymarin, as an absolutely safe drug, has been shown to be effective in reducing oxidative stress and inflammation after CABG and to have reduced the incidence of POAF. In the future, a large multicenter study could confirm the efficacy and safety of silymarin in cardiac surgery patients and influence the eventual routine use of this drug in reducing inflammation and oxidative stress.
